# The After-Care and Employment of Consumptives

**Published:** 1910-11-12

**Authors:** 


### SPECIAL INSTITUTIONAL ARTICLES.

### THE AFTER-CARE AND EMPLOYMENT OF CONSUMPTIVES.

### AN IMPORTANT DISCUSSION.

Under the auspices of the Charity Organisation Association, a conference of ladies and gentlemen interested in the important question of finding suitable employment for, and otherwise taking an interest in, patients who had been undergoing treatment for consumption at a sanatorium, was held on Friday, November 4, at Denison House, Vauxhall Bridge Road, under the presidency of Colonel Montefiore. There was a good attendance, and the subject was approached from various standpoints.

The Chairman explained that the purpose of the meeting was to hold an informal conference of representatives of sanatoria and health societies and the Charity Organisation Society to discuss the after-care and employment of the consumptives discharged from sanatoria. It was a very important subject, and all who had to do with the disease knew how necessary it was to arrive at a conclusion as to the best way of dealing with members of the working classes when they returned from sanatoria.

A letter was read from Dr. Sidney Davies, Medical Officer of Health for Woolwich, pointing out that the good results of sanatorium treatment were often lost owing to the want of after-care, and the lack of work suitable to the patient, so that there was frequently a relapse, thus much of the work spent on sanatoria treatment was wasted. The Charity Organisation Society could do much in the establishment of local after-care committees. He also advocated communication between sanatoria and consumption hospitals, so as to prevent the present overlapping.

#### The Crux of the Problem.

Dr. Jane Walker said that in former days there was a rush to sanatoria of cases which were too ill to benefit by the treatment; they clutched at it, as a drowning man did at a straw. Croakers then said that sanatorium treatment was not successful. But experience showed that a large percentage of cases of phthisis were curable. Especially in the case of the poor, the process of cure did not end at the sanatorium. That institution must be regarded as an educational establishment for teaching sufferers from consumption how to live their lives to the best advantage to themselves and their fellows. Patients in the well-managed sanatoria for working classes were not allowed to loaf about and do nothing; it was not good for them or for their disease. Notwithstanding the system in vogue at Kelling, an energetic patient recently stigmatised the institution as a "school for idleness." The work done at a sanatorium could be divided into that which occupied the patient during his stay, and that which fitted him for his work outside. The first was gradually regulated, and was an agency in the recovery; but the second class was the more difficult, namely, that directed to fit the patients for earning their living afterwards. It often happened that the best work discharged patients could do was that which they were doing before, and in that case their anxiety was reduced to a minimum. But many employers refused to take back those who had had a breakdown due to the disease; and the worst offenders in this respect were Government departments, the Navy being the worst of all. The sailor, catching tuberculosis in the Service, was invalided out without an attempt being made to make him well, and with practically no means with which to start him again. Often the doctor said to a consumptive patient, "You must leave London and find work in the country." That was often bad advice, because impracticable. People could stand bad air and poor surroundings for a part of the day if their leisure and nights were spent in good conditions. An unsuitable occupation with plenty of money was better than an ideal situation with very little coming in. In deciding on the trades taught in sanatoria, regard must be had to those offering the greatest payment. For men, motor-driving was an excellent occupation. French and ordinary gardening were also good. There was a demand for laundry work, but such work was fraught with difficulties. It could not be too strongly insisted that however well a patient might be when he left the sanatorium, he needed careful watching for at least two years after leaving. The cases among the poor who had done best were those which had been followed up and looked after for a long time. Lack of good food and good surroundings would be almost sure to result in a relapse. She suggested that where there was a tuberculosis dispensary in the neighbourhood, a patient should report himself and a visitor should look after him periodically. Labour exchanges might also help. In answer to the statement that many able-bodied people could not get work, the speaker declared that consumptives were amongst the most skilful people, who did much for the good of the State. Men's cases were far harder than women's. Some organisation or society in the neighbourhood or town to which the patient would go should know of his or her discharge, and should have a sympathetic friend. The self-respecting working man would not cadge, and anyone helping him would be well repaid. Any scheme should be national; often the State might do more than it did.

#### Some Facts and Figures.

Dr. Burton Fanning (Kelling Sanatorium, Norwich) said he thought it would be of interest, in view of the present discussion, to go through the records of the sanatorium with which he was connected, to ascertain the effects of various occupations on the subsequent health of patients who had been discharged from the institution. He agreed with every word which Dr. Jane Walker had just uttered, and his figures supported her contentions. They did not lose sight of more than 1 per cent. of their ex-patients. About 1,000 patients had passed through the Sanatorium, but he would deal only with those whose disease on arrival was mild up to 1903; 306 of those were available for the inquiry—205 men, 101 women. 123 of the men and 72 of the women returned to their original occupations, or 60 per cent.; 31 per cent. went to new occupations; 8 per cent. kept out of work. For the latter small figure one probably had to thank the After-Care Committee, about which Dr. McConnel would speak. Yet it did seem to support Dr. Jane Walker's statement that consumption seemed to attack the salt of the earth. The figures also came out in an interesting way concerning the effects of indoor and outdoor employment. Of those who had gone to outdoor work, 76 per cent. had kept well, 12 per cent. fairly well, 10 per cent. had become worse. Of those who had gone back to indoor work, 73 per cent. had kept well,

11 per cent. fairly well, while 15 per cent. had got worse. The difference was thus in favour of those who had returned to outdoor work; 12 of the number, who had previously been employed indoors, were put on to hard work on the land, and they had all kept well. The number was small, but even so the fact was significant. Carefully regulated work was an important part of the treatment of tuberculous patients, and hard work seemed important also in keeping the patient well afterwards. But it was clear that the work chosen for consumptives should not be very laborious; the work should be carefully selected and supervised. The C.O.S. would be filling a very great gap if it could institute centres in every place where ex-patients could be handed over to be looked after. It had been found almost impossible to get large employers of labour to take ex-consumptives; but many employers were very charitable to their old servants who had had the disease, and took them back under better conditions, such as shorter hours and lighter work.

### Self-Help, Co-operation, and Inquiry.

Dr. Ethel Carling considered that the question should be one of co-operation. There was an awakening campaign all over the country concerning tuberculosis, and it was desirable to switch on to that general campaign the question of after-care. She would like to see operative in this work a system which had proved so valuable in hospitals, namely, a system of almoners. In the case of consumptives, the whole question of the life of the patient had to be faced if the matter was to be dealt with satisfactorily. To further that idea, it would be of the greatest help to have a journal which would be a medium for the exchange of ideas, perhaps something in the form of a parish magazine, in which could be published the names of those who were keen on the subject in various parts of England. It would also enable ex-patients to maintain their interest in the sanatoria which had done so much for them. Ex-patients made excellent missionaries, because their experience made them very keen on the tuberculosis campaign. Where there was a well-established C.O.S. one knew where to go to, but there were many places without a C.O.S., and it was then difficult to know whom to go to befriend the ex-patients. She was glad to know that Dr. Davies, after repeated applications, had at length induced the Woolwich Arsenal authorities to give most advantageous terms of re-instatement to those who had been to a sanatorium. One result of that was, that men were not now afraid, as they formerly were, of applying to the doctor for relief at an early stage of the disease. Formerly cases received into the sanatorium from Woolwich Arsenal were left much too late, but now they were in a much more hopeful stage on arrival for treatment.

### Do Sanatoria Manufacture Drones?

Dr. McConnel (Kelling Sanatorium), said that owing to lack of funds on one hand, and the urgent need to get home again on the other, the time under treatment was often too short to effect a permanent cure or arrest of the disease. That short stay made after-care all the more necessary. An effort was made to accept only early cases, and the aim was to render them fit for work again. This was impressed upon the patients. If patients did nothing but rest, feed, and take endless walks, they got out of hand, and they left the institution with the idea that they were delicate and could not work, and so became a nuisance to themselves and their friends. At Kelling there was a special officer, part of whose duty it was to find work of a suitable character for the patients when they left the sanatorium. They were kept as busy as possible, so that if they were suitable cases they did not develop into loafers. The institution was able to keep on ten patients for after-care, and this number would be increased to fifteen shortly. Some stayed on and became part of the permanent establishment. The statistics showed that this procedure was very satisfactory. With regard to assistance and advice about a future career, a definite routine was followed. Every case entering the sanatorium was examined and investigated a month after admission; by which time the prognosis could be assumed and a suitable line of work for the patient decided upon. Many patients were able to return to their former occupations, and this was encouraged, because experience showed it entailed less anxiety than taking up a new occupation. Each patient was advised to have a bed and bedroom to himself, and tuberculous parents were instructed to bring up their children to some career which gave them a good chance of resisting the disease; it was not fair to leave predisposed children to take their chance in unhygienic surroundings: they must have fresh air, good food, and sufficient rest before and after meals. By this means those persons became, in turn, educators of others. The after-care Dr. McConnel considered of the utmost importance, but it entailed more work than the ordinary sanatorium staff could give. At Kelling they did what they could in this respect. Christmas cards and letters were used to keep in touch with ex-patients and to ascertain their condition. Only about four per cent. failed to reply. Invitations were given to former patients to attend and report themselves periodically. They were then given fresh advice. But it should not be regarded as the duty of sanatoria to look after patients after they left. Employers and friends who sent the patients to the sanatorium should be asked to help in the matter. If after sanatorium treatment cases were allowed to relapse from want of after-care, the money spent in treatment was largely wasted. All sanatoria should have After-care Committees, but their field of work must necessarily be limited to the time the patient was at the institution: the subsequent after-care should devolve upon the charities and friends who sent the patients.

### Lessons from Frimley.

Dr. Paterson (Frimley) said there were not many people who knew what happened to patients after they left the sanatorium; but the only solution of the problem was that which had been advocated, namely, to send the patients back to their previous occupation, for at that they would get the best remuneration. The profession had too often advised patients to give up their occupation and get out-door work; but that was not good advice, for they would earn too little and so practically starve, which greatly conduced to a physical breakdown. One would think that a man who had had tuberculosis must never put his nose inside a house again. If there was to be no means of continuing the care of the patient after he left the sanatorium, the money spent in treatment might almost as well be thrown into the Thames. The extreme rest-cure after tuberculosis was also bad. Dr. Paterson narrated the case of an ex-patient, a sailor, who could not get work, and who, when in need of a rest, went the round of the various consumption hospitals, knowing quite well what his physical signs were. There had been about 1,700 patients pass through Frimley sanatorium, but he did not know a case of a person getting open-air employment unless he had had previous knowledge of open-air work. There was a type of tuberculosis case which one knew would relapse as soon as he got into the outside world again; and such could be a useful wage-earner if a place kept by Poor-Law authorities could be established.

At present these patients went into the world, spread the infection, and died.

### Questions and Suggestions.

Mr. E. S. Kemp (Secretary, Medical Committee, C.O.S.) said he wished to make one or two suggestions which the C.O.S. would like to hear answered, if possible. He did not wish to dwell on the enormous difficulties always experienced by the District Committees in getting men back to work who had been in a sanatorium. In many cases it was not possible to return to their former occupation. And all the cases did not get treatment early, so that there were very serious ones. Advertising and various other means had been tried, but had failed. Was it possible so to train patients in sanatoria that they could work effectively afterwards? Was there anything open besides motor-driving? Was it possible for sanatoria to find employment in the open labour market for men who had left a sanatorium? Labour exchanges had been mentioned to him, but would the Kelling labour exchange look at a man from another county? That might depend on the man in charge of the exchange, but had it been tried? He agreed that it was the duty of the friends of patients to see to their after-care. not the sanatorium authorities. The most important matter, from the standpoint of the C.O.S., was the question of employment after the patient had returned home. He had been told that a C.O.S. in the North had bought up the window-cleaning for those who had left the sanatoria. On the previous day he was speaking in Whitechapel at a working men's debate on Consumption, and instances were given of the kinds of work which some returned patients undertook. Another suggestion he had heard was that a conference of more or less benevolent-minded employers might be convened to try to get them to employ some of the unfortunate ex-patients whose cases were now under discussion. It was not only the patient who needed educating, but, in nine cases out of ten, the employer, who often got scared at the idea of consumption.

### State Aid for Consumptives.

Mrs. Pearse related the results obtained by the C.O.S. office at Vauxhall. Eleven cases had been back from sanatoria long enough to enable an idea to be formed of the results. In three the results were good; in seven fairly good, while only one had broken down. As to work, seven returned to good work, while three had never had any regular work since, and one had failed to find any work—the one whose health failed.

Dr. Glover Lyon (Victoria Park Hospital) said he remembered the pre-sanatorium days, when one could be certain that a patient who presented certain signs would, in 99 cases out of 100, be dead in five years. But now the case was very different, for patients had a very good hope of recovery. He referred to the efforts which were being made to provide a sanatorium for Victoria Park Hospital, at the low cost of £80 per bed. He did not think it was sufficiently realised how much Dr. Paterson had done for the treatment of consumption by his system of graduated exercises. Tram-car conducting and driving was a good occupation for ex-patients, as also was motor driving. He believed it was the duty of the State to interfere in such a matter. If nothing was done for consumptives, they simply came back among the community, who were mean enough to leave them unprovided for, and infected others.

### The Woman's Side.

Miss Curtis (Superintendent, District Nurses, Hammersmith) pointed out the difficulties attending the case of the consumptive woman, especially the domestic, whose work was not, in nine cases out of ten, open to her again. And the married woman often returned to a poverty-stricken home, in which the husband might be out of work. She advocated the registration of ex-patients.

Miss McGaw expressed the hope that one day there would be a working farm colony near London. It was being tried in the North, and was found to help the question of outdoor work for ex-patients. After eighteen months or two years there, they were better able to pass to ordinary farm work. At Paddington Dispensary there was the same difficulty as others had experienced of getting work for ex-patients.

Mrs. Norman Moore suggested that a list of people might be compiled who were willing to employ persons coming from sanatoria.

### Discharged Consumptives as Domestics.

Miss Dalton thought the public should be taught that they ran no risk in employing domestic servants who had been discharged from a sanatorium. Articles contributed to magazines by doctors and others would be a good means of doing this.

Dr. Robert Maguire (Brompton Hospital) said the matter under discussion had concerned the Brompton Hospital authorities for years, and reminded the meeting of the paper written by Dr. Paterson, then connected with Brompton Hospital, and now of Frimley Sanatorium, advocating the sending of discharged patients back to their own work. If an artisan, who may have been earning £4 or £5 a week. were told he must in future work in the country, he was a little dubious as to the reality of his cure. There had been a lamentable scare created in the minds of the public as to the infectiousness of consumption. People should be taught that except in an active stage phthisis was not communicable, especially as the consumptives had themselves been taught wherein the danger lay, and how to avoid it. The work set for ex-patients should not be such as to tax their strength unduly. It was necessary to point out that the scare had gone a great deal too far, and that if the public were educated properly it would not be scared at all.

Dr. Glover Lyon did not think anyone in authority was warranted in saying that a patient who took up work as a housemaid did so without danger of communicating the disease to others.

Dr. Jane Walker replied on the discussion. She wished it to be understood that she was not one who thought that if anything was required to be done the State should do it. But even so, the State was really the public. Every association and agency for dealing with the poor ought to be linked up and act in concert in this matter. The difficulty of getting work for women afterwards was not nearly as great as that for men. It was extremely gratifying to hear about the preferential terms of re-employment granted to employees of Woolwich Arsenal on their return from sanatoria. The public had not been over-educated on the question, though it might be said by some that it had been wrongly educated.

The Chairman offered thanks to all who had taken part in the discussion, especially to Dr. Jane Walker for her opening speech. The Medical Committee of the C.O.S. would have the full report of the meeting, and would make the best use of such an excellent conference.

